# Impact of DREAMS interventions on attitudes towards gender norms among adolescent girls and young women: Findings from a prospective cohort in Kenya

**DOI:** 10.1371/journal.pgph.0002929

**Published:** 2024-03-06

**Authors:** Kate Andrews Nelson, Faith Magut, Sarah Mulwa, Jane Osindo, Vivienne Kamire, Sammy Khagayi, Julie Pulerwitz, Sarah Cook, Annabelle Gourlay, Abdhalah Ziraba, Daniel Kwaro, Sian Floyd, Isolde Birdthistle

**Affiliations:** 1 Faculty of Epidemiology and Population Health, London School of Hygiene & Tropical Medicine, London, United Kingdom; 2 Kenya Medical Research Institute, Kisumu, Kenya; 3 African Population and Health Research Center, Nairobi, Kenya; 4 Population Council, Washington, D.C., United States of America; 5 National Heart and Lung Institute, Imperial College London, London, United Kingdom; NYU Grossman School of Medicine: New York University School of Medicine, UNITED STATES

## Abstract

The DREAMS partnership aims to deliver a comprehensive package to reduce HIV incidence among adolescent girls and young women (AGYW), including through shifting gender norms. We evaluate DREAMS’ effect on attitudes towards gender norms in two Kenyan settings. AGYW aged 15–22 in Nairobi (n = 852) and Gem (n = 761) were randomly selected for cohort enrolment in 2017–18 and followed-up to 2019. We described the proportion of AGYW and their male peers with equitable attitudes towards gender norms, using an adapted version of the GEM scale. We estimated the association between self-reported invitation to DREAMS (in 2017–18) and AGYW’s attitudes towards two dimensions of gender norms, and then applied a causal inference framework to estimate the difference in the proportion of AGYW with equitable attitudes under the counterfactual scenarios that all versus none were DREAMS beneficiaries. We estimated that overall, 90.2% versus 87.1% of AGYW would have equitable norms around sexual and reproductive health decision-making in Nairobi if all versus none were DREAMS beneficiaries (+3.1; 95%CI:-2.5, +9.0). In Gem, we estimated a risk difference of +1.0 (89.6% vs 88.6%, 95%CI: -3.6,+5.6). There was no evidence for an effect of DREAMS on attitudes towards violence-related norms (Nairobi: 82.7% vs 82.2%, +0.5; 95%CI: -5.3,+6.5; Gem: 44.3% vs 48.2%, -3.9; 95%CI: -11.7,+3.0). We found no evidence of an impact of DREAMS invitation on individual attitudes towards gender norms. In some cases, equitable attitudes at enrolment left limited scope for improvement, and additional effort may be required to shift inequitable violence attitudes among both AGYW and their male peers.

## Introduction

Adolescent girls and young women (AGYW) in sub-Saharan Africa remain at high, disproportionate risk of HIV infection, driven in part by structural factors and the overlapping vulnerabilities these factors create [1, 2]. Recognising these vulnerabilities, PEPFAR and private-sector partners launched the “DREAMS Partnership” in 2015 to support AGYW in leading “Determined, Resilient, Empowered, AIDS-free, Mentored, and Safe” lives. A central component of DREAMS is the intention to deliver a package of interventions, resulting in a layering of services designed to simultaneously address the biological, behavioural, and social determinants of HIV risk [3].

One way DREAMS aims to reduce HIV incidence is by shifting gender norms, which can be defined as collective beliefs about the appropriate behaviours of men and women. Gender norms shape sexual and reproductive health (SRH) exposures, behaviours, and risks [4] and inequitable norms can negatively impact health by influencing actions such as condom and contraceptive use, sexual debut and intimate partner violence [5–7].

The DREAMS “core package” includes a set of interventions delivered at both the individual and contextual levels [3]. Multiple interventions in the DREAMS “core package” seek to influence norms, including social asset building and violence reduction programmes for AGYW and their male peers; school-based programmes; and community strengthening efforts targeted to partners, parents, and caregivers [8]. As appreciation for the importance of social norms in health has grown, more programmes have sought to incorporate gender-transformative components (e.g. SASA [9], Stepping Stones [10], Tsima [11], Program H [12]). This has led to an increasing need to build an understanding of what works to shift complex gender norms.

Cislaghi and Heise’s dynamic framework for social change builds on Bronfenbrenner’s socioecological model and conceptualizes behaviour as influenced by four overlapping domains: individual, social, institutional, and material [13, 14]. A version of the framework tailored to adolescent sexual and reproductive health (SRH) places power and social norms at the centre, highlighting the multi-level relationships between norms and these domains at each intersection [15]. For example, between the individual and social domains, individuals may internalise norms by observing both what others *do* and by understanding what others *expect* [16, 17].

The relationships between community-level norms and individual-level behaviour pose evaluation challenges, and there is ongoing discussion about what each level of measurement contributes. One approach is to measure attitudes at the level of the individual, to gain insight into gender norms prevalent in their communities. A tool often used is the Gender Equitable Men (GEM) Scale [18], designed to capture individuals’ gender norms attitudes at a point in time. While the scale was developed for use with young men, it has since been applied in many contexts, including among AGYW [19].

In this study, we evaluate the impact of DREAMS on attitudes towards gender norms among AGYW. Using GEM Scale data collected in urban and rural Kenya as part of an independent evaluation of DREAMS, we describe attitudes towards different dimensions of gender norms among AGYW and their male peers. We then estimate the effect of DREAMS on AGYW’s attitudes approximately three years after programme implementation.

## Methods

### Study design

The DREAMS impact evaluation makes use of existing demographic surveillance platforms: the Nairobi Urban Health and Demographic Surveillance System in two informal settlements, and the Kenya Medical Research Institute (KEMRI)/CDC site in Gem, a rural area in western Kenya [20, 21]. AGYW residing in these sites were randomly selected from population-wide sampling frames for enrolment into age-stratified cohorts.

In Nairobi, AGYW aged 10–14, 15–17 and 18–22 years were enrolled in March-July 2017, and in Gem, AGYW aged 13–17 and 18–22 years were enrolled in January-October 2018. Participants were followed up in July-December 2018 and May-August 2019 in Nairobi; and January-November 2019 in Gem. Multiple revisits were made to attempt to reach all eligible participants. Participants under age 15 are not included in this analysis due to differences in survey items. DREAMS-specific modules were also incorporated into the cross-sectional general population surveys in Nairobi, which we use to describe gender norms among young men.

Data were collected electronically by researchers through in-person interviews. Questionnaires included items on sociodemographics (including self-assessed household poverty and a household asset index), sexual health and behaviour, gender attitudes, social support, and experience of violence. Participants also reported their awareness of DREAMS and participation in DREAMS services. Additional details on the study design and data collection procedures have been published previously [22].

#### Dreams interventions

The DREAMS core package aimed to empower AGYW to reduce their risk, strengthen families, mobilize communities for change, and reduce risk among sexual partners. At the individual level, this included: HIV testing services for AGYW and male partners, social asset building interventions, expanding the availability and range of contraceptives, condom promotion and provision, school-based SRH curricula, targeted provision of pre-exposure prophylaxis, and post-violence care. At the contextual level, interventions included: social protection interventions for AGYW and their caregivers (such as educational subsidies), parenting programmes on adolescent SRH, community-based mobilisation and norms-change messaging, and efforts to characterise male sex partners to target interventions [8]. Invitation to DREAMS was not randomised but targeted to the most vulnerable AGYW, identified using the ‘Girl Roster’ tool [23].

Staggered rollout of DREAMS interventions began in 2016, with delivery led by implementing partners in each site. While implementation began prior to cohort enrolment, the package took time to roll out. New services, including community-based norms programming, took longer to introduce [8, 24]. All interventions were being delivered by 2017.

#### Measurement of DREAMS exposure and gender norms outcomes

We used self-reported invitation to DREAMS (yes/no) by 2018 to define exposure. AGYW invited in 2017 and/or 2018 were considered beneficiaries, irrespective of services accessed. This definition emulates an “intention to treat” approach, reflecting real-world variation in uptake of the intervention package [25]. To complement this approach, we also present a “per-protocol” analysis, defining DREAMS exposure as accessing 3 or more interventions. Awareness and uptake of DREAMS interventions among intended AGYW beneficiaries was high, with variation by age group and intervention category, and has been described elsewhere [8]. For example in Nairobi, >80% of AGYW invited accessed at least two primary interventions but few accessed all seven.

Individual attitudes towards gender norms were measured using items from the GEM Scale (S1 Table), covering three of four original GEM domains and additional items from adaptations in other settings [18]. Participants reported their level of agreement with each statement on four levels: “strongly agree,” agree,” “disagree,” “strongly disagree,” or alternatively, “don’t know.” In Gem, Kenya, six items had fewer response options: “agree,” “disagree,” or “don’t know.” Some statements were equitably-phrased (“*A couple should decide together if they want to have children*”), whereas others were inequitably-phrased (“*There are times when a woman deserves to be beaten*”). Previous work suggests the scale measures two dimensions of gender norms, equitable and inequitable, aligned with the item phrasing, and subsequent studies have used alternative groupings of the items to measure different dimensions of gender norms [26].

We validated this modified GEM scale using exploratory and confirmatory factor analysis, described in S1 Text [27]. The dimensions of gender norms identified by the best-fitting model were taken as outcomes, provided they had adequate internal reliability (ordinal alpha > = 0.70). This resulted in two dimensions, capturing attitudes to norms around i) SRH decision-making and ii) violence (Table 1).

**Table 1:** GEM Scale items used to measure each dimension of attitudes towards gender norms.

GEM Scale items
***SRH decision-making norms***
A couple should decide together if they want to have children.
In my opinion, a woman can suggest using condoms just like a man can.
If a man gets a woman pregnant, the child is the responsibility of both.
A man and a woman should decide together what type of contraceptive to use.
A man and woman should decide together whether to use a condom.
A man should know what his partner likes during sex.
***Violence norms***
There are times when a woman deserves to be beaten.
A woman should tolerate violence in order to keep her family together.
If a woman cheats on a man, it is okay for him to hit her.
If someone insults a man he should defend his reputation with force if he has to.
A man should be outraged if his wife/partner asks him to use a condom
It is okay for a man to hit his wife if she won’t have sex with him.

Participants’ scores for each dimension were calculated by taking the mean of their non-missing responses, such that higher scores indicated more equitable attitudes. Participants who answered “don’t know” or refused more than one third of items for a factor were given a missing score for that factor. Otherwise, “don’t know” was coded as neutral, 2.5. Scores were dichotomized using a cut-off of 3 or greater to reflect a hypothesised qualitative difference between mean agreement and mean disagreement, resulting in a binary outcome measure defined as yes or no for equitable attitudes. We also conducted a sensitivity analysis using participants’ GEM score as a continuous outcome. The same approach and items were used to calculate scores for young men in Nairobi, to support comparability.

### Statistical analysis

For each site, we constructed a directed acyclic graph (DAG) to represent the hypothesised causal relationship between DREAMS invitation, attitudes towards gender norms, and other measured characteristics (S1 Fig). The DAGs were informed by the targeting of DREAMS invitation, existing literature, and our understanding of the contexts.

We described AGYW’s characteristics at enrolment by age group, setting, and DREAMS invitation status. We also summarised the mean response to each GEM scale item in 2019. To contextualise AGYW’s attitudes, we compared their outcomes over time, from enrolment (2017 or 2018) to 2019, and to the same outcomes among men aged 15–24 in 2018 and 2019.

To estimate the impact of DREAMS on AGYW’s attitudes, we first used logistic regression to estimate the associations between being a DREAMS beneficiary and attitudes towards gender norms. We constructed unadjusted, site- (in Nairobi) and age group-adjusted odds ratios (ORs) and 95% confidence intervals (CIs) before including potential confounders identified through the DAGs in a multivariable model.

The causal effect of DREAMS was then estimated using propensity score logistic regression adjustment, with separate analyses for the two settings. The propensity score is defined as an individual’s probability of being a DREAMS beneficiary, given their individual and household characteristics. Scores were estimated using logistic regression, with DREAMS invitation (by 2018) as the outcome and the potential confounders (at enrolment) as explanatory variables.

Next, for each equitable norms outcome, we fit a logistic regression model with age group and the estimated propensity to be a DREAMS beneficiary as explanatory variables. This was done separately for DREAMS beneficiaries and non-beneficiaries. These two logistic regression models were then used to predict the expected proportions of AGYW with the outcome, under the counterfactual scenarios that non-DREAMS beneficiaries were all DREAMS beneficiaries and vice-versa. The effect of DREAMS was estimated as the difference between these two predicted proportions, and 95% CIs were generated using bootstrapping. This approach has been described previously [25].

Analyses were done in Stata 16, and R (v4.1.0) using the lavaan package (v0.6) [28–30]. The latter was used for the scale validation and factor analysis presented in S1 Text. DAGs were created using DAGitty [31].

### Sensitivity analyses and assumptions

To evaluate the robustness of our estimates, we applied three alternative techniques: propensity score stratification, inverse-probability-of-treatment weighting (with the probability of treatment equal to the propensity score), and also using predictions (of the proportion with the outcome of equitable attitudes) derived from a multivariable logistic regression model of the outcome variable on the potential confounding variables.

In order to interpret our results as causal estimates of the effect of DREAMS invitation, certain assumptions must hold including positivity, conditional exchangeability, consistency, and no interference [25]. We consider each of these further in the discussion.

### Ethics

Ethics approvals were obtained from AMREF Health Africa, KEMRI, and the London School of Hygiene & Tropical Medicine. All participants provided written informed consent, and parent/guardian consent was obtained for those under age 18. Additional information regarding considerations specific to inclusivity in global research is included in S1 Checklist.

## Results

### Sample characteristics

In Nairobi, 1081 AGYW aged 15–22 were enrolled in 2017, of which 852 (79%) were retained to 2019. In Gem, 888 AGYW aged 15–22 were enrolled and 761 (86%) were followed up. By 2018, 73% (n = 628) of the cohort in Nairobi and 56% (n = 429) in Gem had been invited to DREAMS. Retention in the cohort in 2019 was higher among those in versus out of school at enrolment, those invited to DREAMS, and those who had never had sex, and has been described in detail previously [32].

In Gem, AGYW invited were somewhat less likely to have had sex, more likely to be food insecure, and had lower socioeconomic position as compared to those not invited (Table 2). In Nairobi, DREAMS beneficiaries were relatively younger, less likely to have been married or pregnant, and more likely to be food insecure.

**Table 2:** a, b. Characteristics at enrolment among AGYW followed up in 2019, by DREAMS invitation status, in Gem (2a) and Nairobi (2b).

2a. Gem, Kenya (enrolment in 2018)			
		**DREAMS beneficiary status**	
**Characteristics at enrolment**	**Overall** (N = 761)	**Never invited** (N = 332)	**Invited by 2018** (N = 429)
**Age group at enrolment**	**n (%)**	**n (%)**	**n (%)**
15–17	365 (48.0)	157 (47.3)	208 (48.5)
18–19	209 (27.5)	80 (24.1)	129 (30.1)
20–22	187 (24.6)	95 (28.6)	92 (21.4)
**Highest education level completed**			
None or primary	248 (32.6)	106 (31.9)	142 (33.1)
Secondary or higher	351 (46.1)	135 (40.7)	216 (50.3)
Unknown	162 (21.3)	91 (27.4)	71 (16.6)
**Composite sex and pregnancy history**			
Never had sex	457 (60.1)	182 (54.8)	275 (64.1)
Ever had sex, never pregnant	145 (19.1)	69 (20.8)	76 (17.7)
Ever pregnant	159 (20.9)	81 (24.4)	78 (18.2)
**Orphanhood status**			
Not an orphan	449 (59.0)	191 (57.5)	258 (60.1)
Single or double orphan	126 (16.5)	53 (15.9)	73 (17.0)
Unknown	186 (24.4)	88 (26.5)	98 (22.8)
**Food insecure**			
No	585 (76.9)	276 (83.1)	309 (72.0)
Yes	176 (23.1)	56 (16.9)	120 (28.0)
**Wealth quantile**			
Poor	312 (41.0)	110 (33.1)	202 (47.1)
Medium	143 (18.8)	60 (18.1)	83 (19.3)
Wealthy	306 (40.2)	162 (48.8)	144 (33.6)
**Self-assessed household poverty**			
Very poor	85 (11.2)	28 (8.4)	57 (13.3)
Moderately poor	554 (72.8)	239 (72.0)	315 (73.4)
Not poor	122 (16.0)	65 (19.6)	57 (13.3)
**Attitudes towards SRH norms (2018)**			
Inequitable	167 (21.9)	64 (19.3)	103 (24.0)
Equitable	594 (78.1)	268 (80.7)	326 (76.0)
**Attitudes towards violence norms (2018)**			
Inequitable	444 (58.3)	197 (59.3)	247 (57.7)
Equitable	317 (41.7)	135 (40.7)	182 (42.4)
2b. Nairobi, Kenya (enrolment in 2017)			
		**DREAMS beneficiary status**	
**Characteristics at enrolment**	**Overall** (N = 852)	**Never invited** (N = 224)	**Invited by 2018** (N = 628)
**Age group at enrolment**	**n (%)**	**n (%)**	**n (%)**
15–17	464 (54.5)	95 (42.4)	369 (58.8)
18–19	181 (21.2)	52 (23.2)	129 (20.5)
20–22	207 (24.3)	77 (34.4)	130 (20.7)
**Site**			
Korogocho	513 (60.2)	143 (63.8)	370 (58.9)
Viwandani	339 (39.8)	81 (36.2)	258 (41.1)
**Currently in school**			
No	312 (36.6)	109 (48.7)	203 (32.3)
Yes	540 (63.4)	115 (51.3)	425 (67.7)
**Highest education level completed**			
None or incomplete primary	92 (10.8)	30 (13.4)	62 (9.9)
Complete primary	170 (20.0)	54 (24.1)	116 (18.5)
Some secondary	410 (48.1)	76 (33.9)	334 (53.2)
Complete secondary or tertiary	180 (21.1)	64 (28.6)	116 (18.5)
**Marital status**			
Never married	695 (81.6)	161 (71.9)	534 (85.0)
Previously (currently) married/ living with partner	157 (18.4)	63 (28.1)	94 (15.0)
**Pregnancy and child history**			
Never pregnant, no children	671 (78.8)	160 (71.4)	511 (81.4)
One or more children	181 (21.2)	64 (28.6)	117 (18.6)
**Orphanhood**			
Not an orphan	664 (77.8)	170 (75.9)	493 (78.5)
Single/double orphan	189 (22.2)	54 (24.1)	135 (21.5)
**Food insecure**			
No	564 (66.2)	166 (74.1)	398 (63.4)
Yes	288 (33.8)	58 (25.9)	230 (36.6)
**Wealth quantile**			
Poor	303 (35.6)	77 (34.4)	226 (36.0)
Medium	277 (32.5)	79 (35.3)	198 (31.5)
Wealthy	272 (31.9)	68 (30.4)	204 (32.5)
**Self-assessed household poverty**			
Very poor	115 (13.5)	23 (10.3)	92 (14.6)
Moderately poor	672 (78.9)	180 (80.4)	492 (78.3)
Not poor	65 (7.6)	21 (9.4)	44 (7.0)
**Attitudes towards SRH norms (2017)**			
Inequitable	72 (8.5)	26 (11.8)	46 (7.4)
Equitable	771 (91.5)	194 (88.2)	577 (92.6)
**Attitudes towards violence norms (2017)**			
Inequitable	287 (33.4)	94 (42.2)	193 (30.8)
Equitable	562 (66.2)	129 (57.9)	433 (69.2)

Footnote: SRH = sexual and reproductive health; wealth quantile derived from a household asset index

#### Attitudes towards gender norms

**Fig 1:**
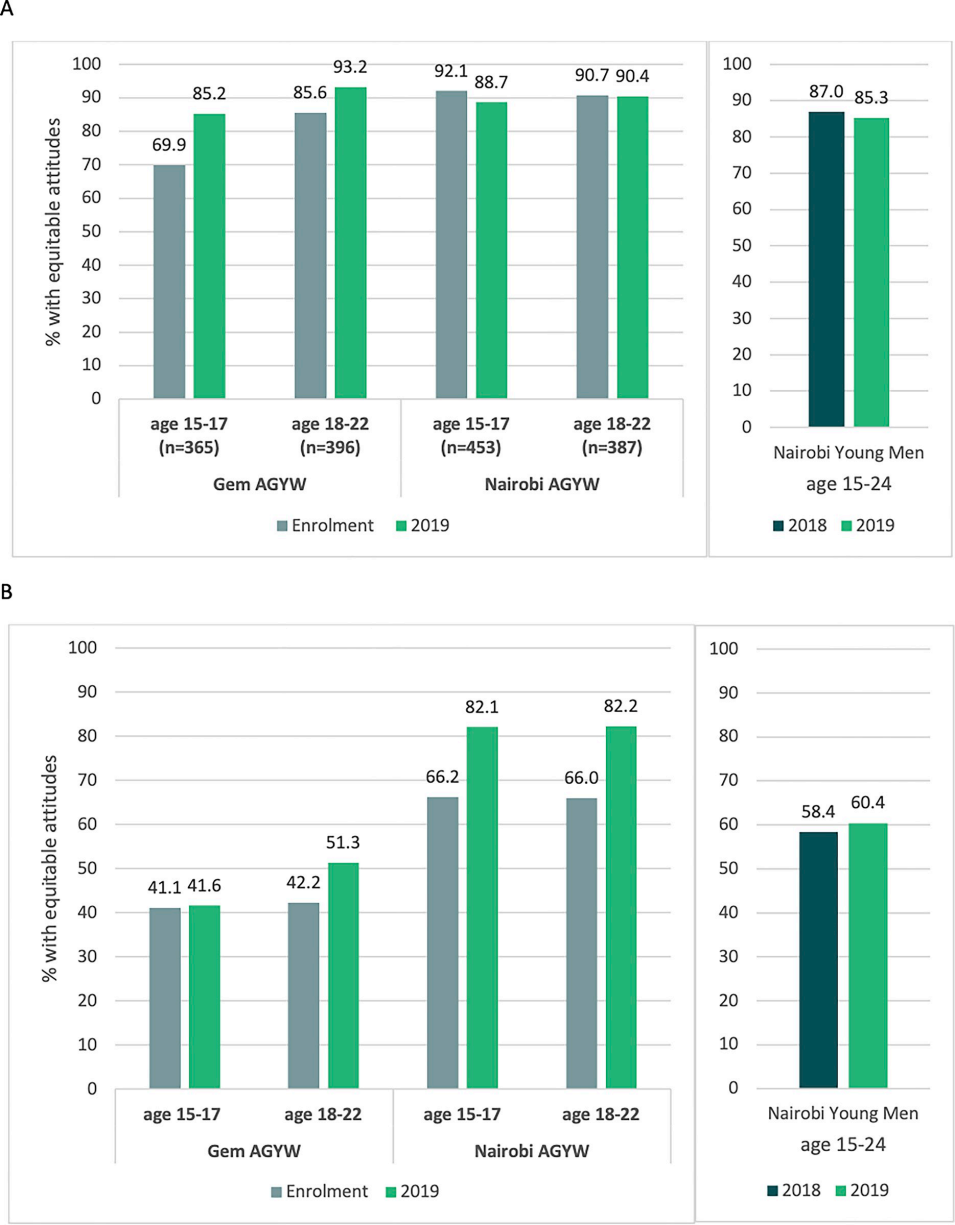
AGYW attitudes towards gender norms at enrolment (2017 or 2018) and in 2019, stratified by setting and age group and compared to attitudes among young men in Nairobi. **1A.** Percent of AGYW participants with equitable attitudes towards **SRH decision-making norms** in Gem and Nairobi (left) and of young male participants in Nairobi (right). **1B.** Percent of AGYW participants with equitable attitudes towards **violence norms** in Gem and Nairobi (left) and of young male participants in Nairobi (right).

Fig 1 shows the overall proportion of participants scoring three or above for each dimension of gender norms (reflecting equitable attitudes). Support for equitable norms around *SRH decision-making* was high among AGYW across both settings and age groups in 2019 (>85%). Among 15–17 year olds, this represented a small increase from 70% at enrolment in Gem, whereas in Nairobi, equitable attitudes were high at both time points (92%, 89%).

In contrast, support for equitable norms around *violence* (indicated by disagreement with inequitable statements) was relatively lower in both settings at enrolment: 41%-42% in Gem and 66% for both age groups in Nairobi. In Gem, these attitudes stayed similar over time among 15–17 year olds (42% by 2019), whereas in Nairobi, equitable attitudes around violence increased substantially over time in both age groups, to 82% in 2019.

In Gem, older AGYW (18–22 years) were more likely to have equitable attitudes than younger participants (15–17): 93% versus 85% for the SRH dimension, and 51% vs 42% for the violence dimension. There were minimal differences between age groups in Nairobi.

Young men’s support for equitable SRH decision-making norms in Nairobi was also high across survey years (87%, n = 2036 in 2018 and 85.3%, n = 1304 in 2019). Demographic characteristics for male participants are presented in S2 Table and in a previous publication [33]. Whereas the proportion of AGYW with equitable attitudes towards the violence dimension increased over time, there was little change between survey arounds among young men (58% vs 60%), leading to a larger gap in 2019 between AGYW and their male peers.

At enrolment, AGYW in Nairobi who were DREAMS beneficiaries had more equitable attitudes towards violence norms than those who were not beneficiaries (Table 2). In Gem, there was a difference among AGWY’s attitudes towards SRH decision-making norms, with DREAMS beneficiaries less likely to have equitable attitudes at enrolment.

### Effect of DREAMS

The associations between DREAMS and attitudes towards gender norms among AGYW in 2019 are shown in Table 3. Support for equitable norms around SRH decision-making was similar amongst DREAMS beneficiaries and non-beneficiaries in Nairobi (90% vs 88%) and Gem (90% vs 89%), and there was no evidence of an association in either setting (Nairobi: aOR 1.3, 95%CI: 0.9–2.2; Gem: aOR 1.1, 95%CI: 0.7–1.9). For the violence dimension, equitable attitudes among DREAMS beneficiaries were slightly higher compared to non-beneficiaries in Nairobi (83% vs 80%) but lower in Gem (44% vs 50%). Again, there was no evidence of an association (Nairobi: aOR 1.1, 95%CI: 0.7–1.6; Gem: aOR 0.8, 95%CI: 0.6–1.1).

**Table 3:** Association between DREAMS invitation and AGYW attitudes towards gender norms in 2019 using multivariable logistic regression, stratified by age group and setting.

	Invited to DREAMS	Total N	n (%) with equitable attitudes	Unadjusted OR (95% CI)	Age and site adjusted OR (95% CI)	Fully adjusted^1^ OR (95% CI)	p value (LRT)
**SRH decision-making norms**							
**Nairobi**							
Overall	No	220	193 (87.7)	1	1	1	
	Yes	620	559 (90.2)	1.3 (0.8,2.1)	1.3 (0.8, 2.1)	1.3 (0.8, 2.2)	0.35
15–17 years	No	91	80 (87.9)	1	1	1	
	Yes	362	332 (89.0)	1.1 (0.5,2.3)	1.1 (0.5, 2.3)	1.1 (0.5, 2.3)	0.87
18–22 years	No	129	113 (87.6)	1	1	1	
	Yes	258	237 (91.9)	1.6 (0.8–3.2)	1.6 (0.8, 3.1)	1.6 (0.7, 3.3)	0.26
**Gem**							
Overall	No	332	294 (88.5)	1	1	1	
	Yes	429	386 (90.0)	1.2 (0.7, 1.8)	1.2 (0.7, 1.9)	1.1 (0.7, 1.9)	0.63
15–17 years	No	157	132 (84.1)	1	1	1	
	Yes	208	179 (86.1)	1.2 (0.7, 2.1)	1.2 (0.7, 2.1)	1.1 (0.6, 2.0)	0.84
18–22 years	No	175	162 (92.6)	1	1	1	
	Yes	221	207 (93.7)	1.2 (0.5, 2.6)	1.2 (0.5, 2.6)	1.3 (0.5, 3.1)	0.60
**Violence-related norms**							
**Nairobi**							
Overall	No	224	178 (79.5)	1	1	1	
	Yes	622	517 (83.1)	1.3 (0.9, 1.9)	1.2 (0.8, 1.8)	1.1 (0.7, 1.6)	0.7
15–17 years	No	95	80 (84.2)	1	1	1	
	Yes	363	296 (81.5)	0.8 (0.5, 1.5)	0.8 (0.5, 1.5)	0.7 (0.4, 1.3)	0.23
18–22 years	No	129	98 (76.0)	1	1	1	
	Yes	259	221 (85.3)	1.8 (1.1, 3.1)	1.8 (1.0, 3.0)	1.6 (0.9, 2.9)	0.10
**Gem**							
Overall	No	332	165 (49.7)	1	1	1	
	Yes	429	190 (44.3)	0.8 (0.6, 1.1)	0.8 (0.6, 1.1)	0.8 (0.6, 1.1)	0.27
15–17 years	No	157	72 (49.5)	1	1	1	
	Yes	208	80 (38.5)	0.7 (0.5, 1.1)	0.7 (0.5, 1.1)	0.8 (0.5, 1.2)	0.29
18–22 years	No	175	93 (53.1)	1	1	1	
	Yes	221	110 (49.8)	0.9 (0.6, 1.3)	0.9 (0.6, 1.3)	0.8 (0.5, 1.3)	0.40

^1^ Adjusted for: age group; educational attainment; marital status (in Nairobi only); sexual experience, pregnancy and child history; socioeconomic position (measured by a household asset index and self-assessed household poverty); food insecurity; and orphanhood; all measured at the time of enrolment.

LRT = Likelihood ratio test; SRH = sexual and reproductive health; CI = confidence interval

Table 4 shows the estimated causal effect of DREAMS on equitable attitudes, stratified by setting. For SRH decision-making norms in Nairobi, we estimated that 90.2% versus 87.1% would have equitable attitudes around SRH decision making if all were DREAMS beneficiaries versus if none were DREAMS beneficiaries (difference +3.1%; 95%CI: -2.5,+9.0%). In Gem, we estimated that 89.6% vs 88.6% would have equitable attitudes around SRH decision making if all were DREAMS beneficiaries versus none were DREAMS beneficiaries (+1.0%; 95%CI: -3.6,+5.6%).

**Table 4:** Estimated causal effect of DREAMS on individual attitudes towards gender norms (equitable vs inequitable) in 2019, using propensity score adjustment.

	% Equitable attitudes in total study population	Estimated % equitable attitudes if none benefit from DREAMS (95% CI)	Estimated % equitable attitudes if all benefit from DREAMS (95% CI)	Risk Difference % (95% CI) *PS-adjusted*
**SRH decision-making norms**				
**Nairobi**				
Overall	89.5	87.1 (81.6, 91.9)	90.2 (87.7, 92.6)	+3.1 (-2.5, +9.0)
15–17 years	88.7	87.1 (78.7, 94.2)	88.9 (85.6, 92.3)	+1.8 (-6.2, +10.9)
18–22 years	90.4	87.2 (80.9, 92.7)	91.7 (87.9, 94.9)	+4.5 (-2.0, +11.6)
**Gem**				
Overall	89.4	88.6 (84.9, 92.0)	89.6 (86.2, 92.5)	+1.0 (-3.6, +5.6)
15–17 years	85.2	84.3 (78.8, 89.8)	85.7 (80.7, 90.5)	+1.4 (-6.0, +9.3)
18–22 years	93.2	92.9 (88.7, 96.4)	93.4 (89.8, 96.4)	+0.5 (-4.6, +5.7)
**Violence-related norms**				
**Nairobi**				
Overall	82.2	82.2 (77.0, 87.2)	82.7 (79.6, 85.7)	+0.51 (-5.3, +6.5)
15–17 years	82.1	85.8 (77.9, 92.1)	81.1 (77.1, 85.0)	-4.8 (-12.3, +3.9)
18–22 years	82.2	78.0 (70.8, 84.7)	84.7 (80.0, 88.9)	+6.7 (-1.3, 14.6)
**Gem**				
Overall	46.7	48.2 (43.1, 53.5)	44.3 (39.6, 49.0)	-3.9 (-11.7, +3.0)
15–17 years	41.6	45.5 (38.1, 53.5)	38.9 (32.0, 46.2)	-6.6 (-16.7, +3.9)
18–22 years	51.3	52.2 (44.9, 60.2)	50.4 (43.6, 56.9)	-1.8 (-12.3, +8.5)

PS = propensity score; CI = confidence interval; SRH = sexual and reproductive health

For equitable norms around violence, DREAMS was estimated to have little impact in Nairobi, with a difference of 82.7% vs 82.2% if all versus none were beneficiaries (+0.5%, 95%CI: -5.3,+6.5%). In Gem, there was a suggestion that DREAMS may have decreased equitable attitudes for the violence dimension. We estimated values of 48.2% and 44.3% (-3.9%, 95%CI: -11.7,+3.0%) with equitable attitudes if none versus if all were DREAMS beneficiaries.

When stratified by age group, there was a suggestion of a positive effect of DREAMS among the older age group in Nairobi, for both dimensions of gender norms. However, there was only weak evidence for this difference (SRH decision-making: risk difference +4.5, 95%CI: -1.99, +11.63; Violence: difference +6.7, 95%CI: -1.3, +14.7).

Results from the sensitivity analyses, including when measuring the outcomes using the continuous GEM scores, were consistent with the findings presented above (Table 5; S3 and S4 Tables).

**Table 5:** Sensitivity analysis: Estimated causal effect of DREAMS on GEM Scale score (continuous) in 2019, using propensity score adjustment.

	Observed mean GEM score*	Estimated mean score if none benefit from DREAMS (95% CI)	Estimated mean score if all benefit from DREAMS (95% CI)	Mean difference (95% CI) *PS-adjusted*
**SRH decision-making norms**				
**Nairobi**				
Overall	3.53	3.54 (3.45, 3.63)	3.53 (3.49, 3.57)	-0.01 (-0.10, +0.09)
15–17 years	3.51	3.56 (3.43, 3.68)	3.49 (3.44, 3.55)	-0.07 (-0.20, +0.08)
18–22 years	3.56	3.52 (3.43, 3.61)	3.57 (3.51, 3.63)	+0.05 (-0.06, +0.16)
**Gem**				
Overall	3.28	3.29 (3.24, 3.34)	3.28 (3.24, 3.32)	-0.01 (-0.07, +0.06)
15–17 years	3.22	3.23 (3.16, 3.29)	3.21 (3.15, 3.26)	-0.02 (-0.11, +0.07)
18–22 years	3.35	3.34 (3.27, 3.41)	3.35 (3.29, 3.41)	+0.01 (-0.08, +0.10)
**Violence-related norms**				
**Nairobi**				
Overall	3.39	3.38 (3.29, 3.46)	3.40 (3.35, 3.45)	+0.02 (-0.07, +0.12)
15–17 years	3.38	3.41 (3.28, 3.54)	3.38 (3.32, 3.44)	-0.04 (-0.18, +0.11)
18–22 years	3.39	3.33 (3.23, 3.43)	3.43 (3.36, 3.49)	+0.10 (-0.03, +0.21)
**Gem**				
Overall	2.92	2.93 (2.89, 2.99)	2.90 (2.86, 2.94)	-0.03 (-0.11, +0.02)
15–17 years	2.87	2.88 (2.82, 2.95)	2.85 (2.79, 2.91)	-0.03 (-0.11, +0.05)
18–22 years	2.97	2.99 (2.91, 3.05)	2.94 (2.88, 3.00)	-0.05 (-0.14, +0.04)

*Possible scores range from 1–4 with higher score indicating more equitable attitudes

PS = propensity score; CI = confidence interval; SRH = sexual and reproductive health

## Discussion

DREAMS is an ambitious, multi-component programme that aims to shift harmful gender norms as one pathway to decreasing HIV risk among AGYW. We evaluated DREAMS’ impact on AGYW’s support for equitable gender norms in two Kenyan settings. We found a high prevalence of equitable attitudes around SRH decision-making across age groups and settings, and a relatively lower prevalence of equitable attitudes around violence norms in Gem compared to Nairobi. In Nairobi, equitable attitudes towards violence norms were also higher among AGYW than their male peers. While there was some shift towards more equitable attitudes over time, we found limited to no evidence for an effect of DREAMS.

For the SRH decision-making norm dimension, high agreement with equitable statements prior to DREAMS may have left limited scope for improvement. This is especially the case in Nairobi, where equitable attitudes were prevalent from enrolment among both AGYW and young men. In Gem, equitable attitudes did increase over time, though this change was not attributable to DREAMS invitation.

In contrast, while equitable attitudes towards violence (disagreement with inequitable statements) increased over time among AGYW in Nairobi, there was little change among AGYW in Gem nor among the young men surveyed in Nairobi. This led to a substantial gap in attitudes between settings by 2019. In Gem, about half of AGYW agreed with inequitable statements around intimate partner violence (e.g. “*It is okay for a man to hit his wife if she won’t have sex with him*”), suggesting a key area for focused interventions. The difference in attitudes between young men and women in Nairobi is also of concern, as inequitable attitudes amongst male peers could lead to AGYW experiencing sanctions from partners or communities should they deviate from a norm.

While DREAMS included contextual interventions targeted to adults and the male partners of young women, there was relatively lower awareness and uptake of DREAMS among these groups [8]. Exposure to community-based interventions (mobilisation, social protection, and parenting/caregiver interventions) among adult men and women was low (<11%) in both sites [8]. Though this study has focused on individual attitudes, norms are inherently relational and present at the intersection of individuals and their communities, resources, and institutions. AGYW’s attitudes may be strongly influenced by those of their peers and caregivers, and involving a broader range of participants may help reach key “change agents” or power-holders who can drive norms change [15].

Where we do see an overall “background” shift towards more equitable norms over time, most notably for SRH decision-making norms in Gem and violence-related norms in Nairobi, it is possible that any effect of DREAMS diffused beyond direct beneficiaries. Case studies have demonstrated that organised diffusion can be an important strategy for encouraging norms change, for example by facilitating information spread through peer groups [34]. While we are unable to detect a diffused effect of DREAMS with this individual-level evaluation, such an effect is likely to take longer to achieve compared to a direct effect on beneficiaries.

A recent review of gender-transformative programmes focused on young people found that encouraging social participation and multi-sectoral approaches involving diverse stakeholders and programming were keys to achieving norms change. Many of the identified programmes (45/61, 74%) were successful in improving health or gender-related indicators, but fewer (16%) showed evidence of broader norms change. They further highlight that although gender norms are often viewed as structural or systemic, most programmes target the individual level, underscoring the challenges of both achieving and measuring norms change [35].

Strengths of this study include our use of a representative sample of AGYW drawn from existing demographic sites in both urban and rural Kenya, with good cohort retention over the study period. The GEM scale performed well in both settings following a rigorous validation approach. While invitation to DREAMS was not randomised, we endeavoured to measure and control for household and individual-level characteristics that may determine DREAMS invitation and attitudes to gender norms. We used a causal inference framework to estimate the impact that can be attributed to DREAMS, and these estimates were robust to alternative methodological approaches, lending confidence to our findings.

We must also consider the validity of the assumptions required to interpret our findings as causal estimates. While we cannot rule out the potential of residual confounding, especially due to the complexity of the intervention and settings, through construction of the DAGs we have sought to consider all potential confounders and minimise bias due to a lack of conditional exchangeability. There is no structural positivity violation as all participants could have potentially received DREAMS; despite intervention targeting, there was always a non-zero probability of participation or non-participation. The consistency assumption means that variants of the exposure do not have different effects on the outcome. Invitation to DREAMS is a well-defined exposure in line with an intention to treat approach. However, this definition masks heterogeneity in individuals’ experiences of the DREAMS package that could differentially impact gender norms and which we did not evaluate [36]. Finally, no interference implies that an AGYW’s attitudes towards gender norms is not influenced by other AGYW’s invitation to DREAMS. It is therefore possible that the observed null effect is partially explained by diffusion of the intervention through AGYW’s social networks, as considered above.

We determined exposure to DREAMS through self-reported invitation which may be subject to misclassification, however most AGYW who reported invitation to DREAMS also accessed interventions [8]. The timing of enrolment 1–2 years after implementation start means that the effects of DREAMS may have been realised prior to enrolment, but the time it took for the DREAMS interventions to be rolled out makes an immediate impact unlikely [24]. We have also measured the prevalence of equitable attitudes towards norms, but this does not directly represent the strength of these norms’ influence over behaviour [37]. This approach is just one of multiple ways to understand the prevailing norms in a community. In some cases, researchers have aggregated attitudes at the community level as a proxy for community level social norms [38, 39]. While individual attitudes and community-level norms may not always align, both offer important insights into how norms may influence health behaviours [37].

This study adds to the growing literature on the potential impact of norms-transformative programmes. Gender norms can have large and wide-ranging consequences for the health and wellbeing of young people, and present numerous challenges for both program implementation and evaluation. Our findings demonstrate some shifts in individual attitudes towards gender norms over time and highlight areas for more focused or inclusive programmes. It is essential for programmes to look beyond individual beneficiaries, including by increasing efforts to reach young men and other members of AGYW’s communities. Though we did not detect a direct effect of DREAMS 2–3 years after implementation, it may take time for the interventions to lead to measurable change in norms. Investments in longer term evaluation efforts could therefore make valuable contributions to the evidence base. By quantitatively describing attitudes to gender norms, we hope to provide insight into areas where programming may be intensified or expanded to address the many factors that influence norms and related behaviours.

## Supporting information

S1 Checklist Inclusivity in global research form. (PDF)

S1 Fig Directed acyclic graphs (DAGs) showing causal pathways and confounding factors for the effect of DREAMS invitation on individual attitudes towards gender norms. (PDF)

S2 Fig Score distributions in 2019 for attitudes towards SRH decision-making and violence norms as measured using the GEM scale, stratified by setting and age group. (PDF)

S1 Table Overview of gender equitable men scale items and the percent of participants responding in support of equitable norms for each item in 2019, stratified by site and age at enrolment. (PDF)

S2 Table Demographic characteristics of young male participants from the general population cohort in Nairobi, by survey year. (PDF)

S3 Table Sensitivity analysis: Estimated causal effect of DREAMS on individual attitudes towards gender norms in 2019, using different analysis approaches. (PDF)

S4 Table Sensitivity analysis: Estimated causal effect of DREAMS intervention access (“per protocol”) on individual attitudes towards gender norms in 2019. (PDF)

S1 Text Validation of GEM scale. A) Standardised factor loadings and factor correlations for an exploratory four-factor model of GEM Scale responses in 2017, among AGYW in Nairobi (n = 892) B) Standardised factor loadings following confirmatory factor analysis in Gem (2018), comparing two and four factor models with and without reducing scale items. C) Scree plot of eigenvalues after exploratory factor analysis (Nairobi 2017).
(PDF)
