# Supplementary material for: Impact of DREAMS interventions on attitudes towards gender norms among adolescent girls and young women: Findings from a prospective cohort in Kenya
Source: PLOS Glob Public Health. 2024 Mar 6;4(3):e0002929. doi: 10.1371/journal.pgph.0002929 (PMC10917282; doi:10.1371/journal.pgph.0002929)

S1 Fig. Directed acyclic graphs (DAGs) showing causal pathways and confounding factors for the effect of DREAMS invitation on individual attitudes towards gender norms.

A. Gem, Kenya DAG

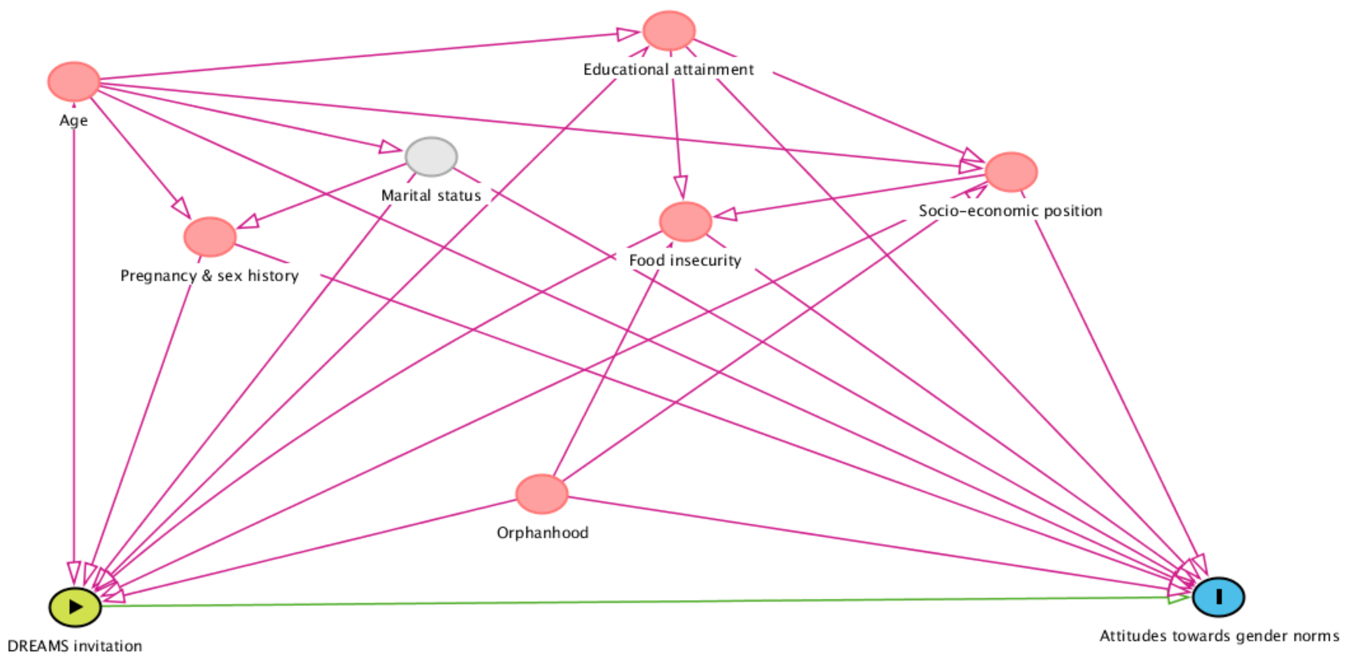

B. Nairobi, Kenya DAG

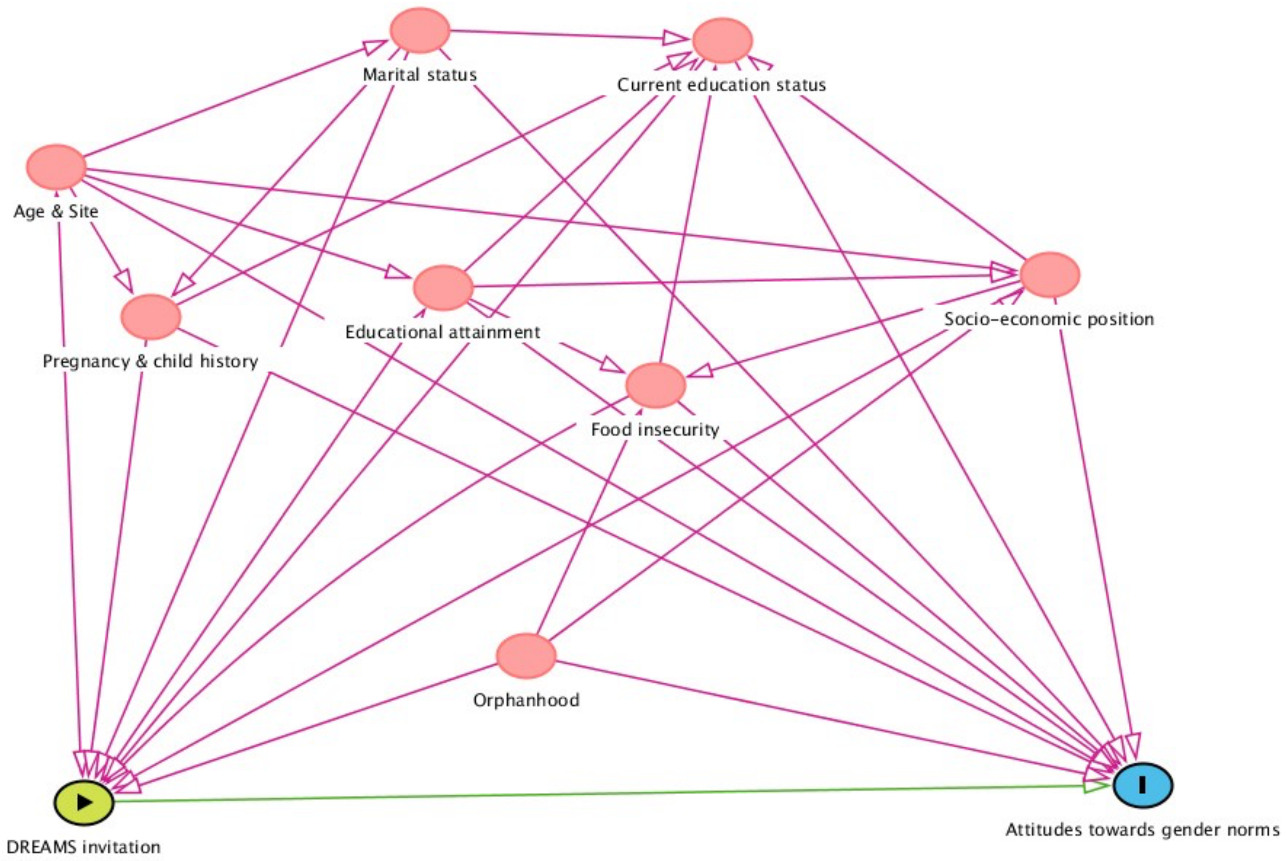

Supplement: S1 Fig — (PDF) [file pgph.0002929.s002.pdf]
