# Supplementary material for: Impact of DREAMS interventions on attitudes towards gender norms among adolescent girls and young women: Findings from a prospective cohort in Kenya
Source: PLOS Glob Public Health. 2024 Mar 6;4(3):e0002929. doi: 10.1371/journal.pgph.0002929 (PMC10917282; doi:10.1371/journal.pgph.0002929)

S2 Figure. Score distributions in 2019 for attitudes towards SRH decision-making and violence norms as measured using the GEM scale, stratified by setting and age group.

a. Score distributions in Gem, Kenya for SRH decision-making (left) and violence (right)

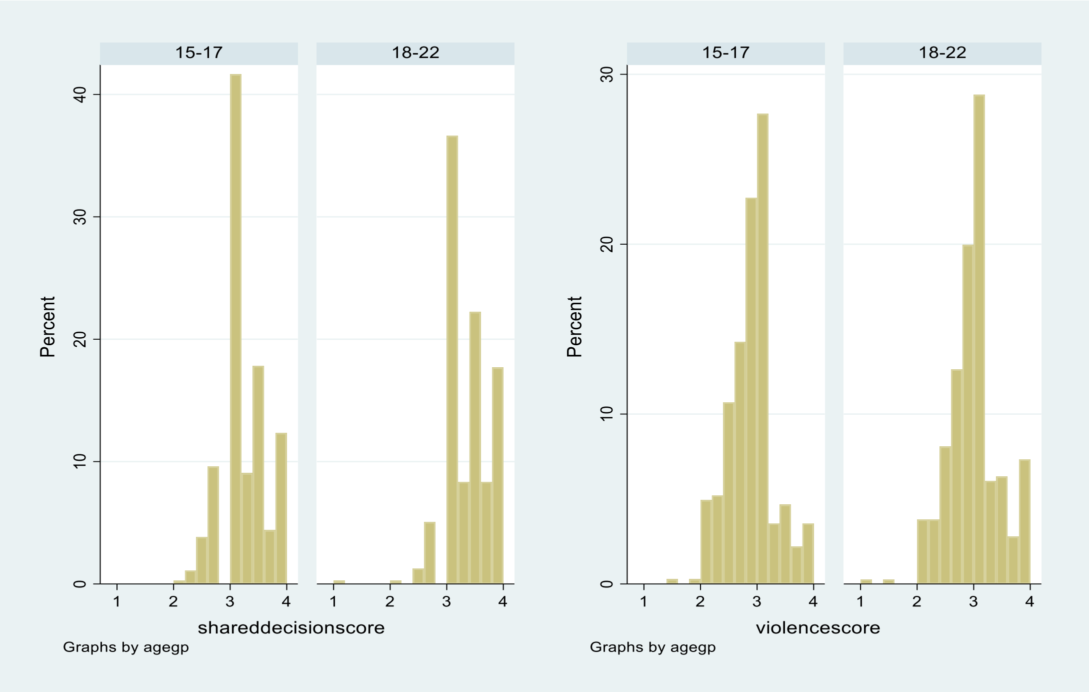

b. Score distributions in Nairobi, Kenya for SRH decision-making (left) and violence (right)

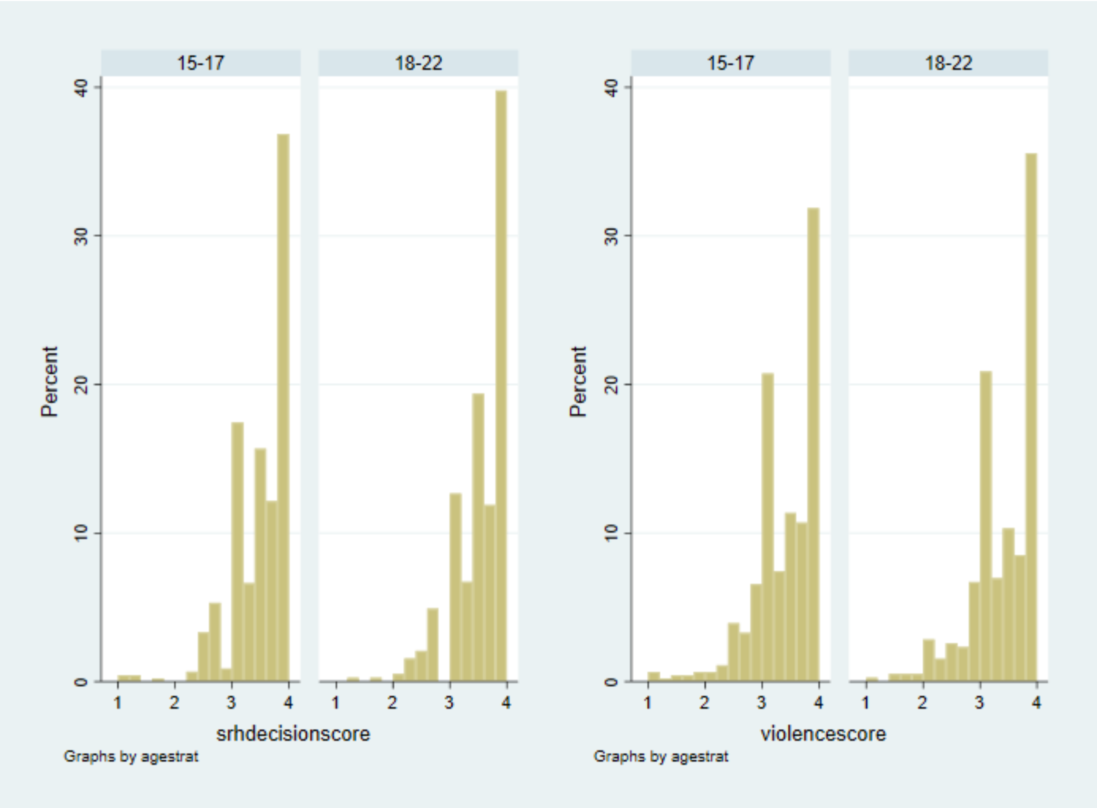

Supplement: S2 Fig — (PDF) [file pgph.0002929.s003.pdf]
