# Supplementary material for: Impact of DREAMS interventions on attitudes towards gender norms among adolescent girls and young women: Findings from a prospective cohort in Kenya
Source: PLOS Glob Public Health. 2024 Mar 6;4(3):e0002929. doi: 10.1371/journal.pgph.0002929 (PMC10917282; doi:10.1371/journal.pgph.0002929)
Supplement: S1 Table — (PDF) [file pgph.0002929.s004.pdf]

S1 Table. Overview of Gender Equitable Men Scale items and the percent of participants responding in support of equitable norms for each item in 2019, stratified by site and age at enrolment.

	**Nairobi, Kenya**			**Gem, Kenya**		
	**15-17**	**18-22**	**Overall**	**15-17**	**18-22**	**Overall**
**Equitably-phrased GEM items**						
A couple should decide together if they want to have children.	94.6	94.1	94.4	98.1	97.5	97.8
If a man gets a woman pregnant, the child is the responsibility of both.	97.8	97.4	97.7	95.9	98.5	97.2
A man and a woman should decide together what type of contraceptive to use.	92.7	93.0	92.8	94.0	98.7	96.5
A man and woman should decide together whether to use a condom. *	92.7	92.5	92.6	90.4	95.7	93.2
In my opinion, a woman can suggest using condoms just like a man can.	90.3	89.4	89.9	82.5	89.4	86.1
In my opinion, a woman can suggest using condoms just like a man can.	81.6	87.6	84.4	84.8	93.4	89.6
**Inequitably-phrased GEM items**						
It is okay for a man to hit his wife if she won't have sex with him.	97.4	95.9	96.7	96.7	95.5	96.1
A man should be outraged if his wife/partner asks him to use a condom	91.0	89.4	90.3	84.4	88.4	86.5
There are times when a woman deserves to be beaten.	92.0	92.5	92.3	78.1	80.3	79.2
If a woman cheats on a man, it is okay for him to hit her.	89.2	85.1	87.3	67.9	72.7	70.4
If someone insults a man he should defend his reputation with force if he has to.	81.7	84.0	82.8	70.1	76.3	73.3
A woman should tolerate violence in order to keep her family together.	77.4	77.1	77.2	56.7	63.6	60.3
You don't talk about sex, you just do it. ◊	87.5	81.1	84.6	89.0	85.4	87.1
It is the man who decides what type of sex to have ◊	86.0	76.0	81.4	84.9	87.1	86.1
Men need sex more than women do ◊	70.3	60.4	65.8	57.0	54.3	55.6
A woman should not initiate sex. *◊	74.6	76.4	75.4	64.9	68.9	67.0
A woman who has sex before she marries does not deserve respect. *◊	76.5	80.9	78.5	68.5	74.2	71.5
Men are always ready to have sex. ◊	58.4	48.2	53.8	62.5	55.3	58.7
A man using violence against his wife is a private matter that shouldn't be discussed outside the couple. *	73.9	74.5	74.2	67.8	64.4	66.5
Women who carry condoms are "easy."	83.8	79.9	82.0	69.6	77.8	73.9
It is a woman's responsibility to avoid getting pregnant.	36.4	39.2	37.7	19.5	24.5	22.1
Only when a woman has a child is she a real woman. *	77.8	76.3	77.1	81.1	83.8	82.5
A real man produces a male child. *	92.5	91.2	91.9	96.2	96.5	96.3

* items not included in original scale, ◊ 2-level option in Gem
