# Supplementary material for: Impact of DREAMS interventions on attitudes towards gender norms among adolescent girls and young women: Findings from a prospective cohort in Kenya
Source: PLOS Glob Public Health. 2024 Mar 6;4(3):e0002929. doi: 10.1371/journal.pgph.0002929 (PMC10917282; doi:10.1371/journal.pgph.0002929)
Supplement: S2 Table — (PDF) [file pgph.0002929.s005.pdf]

S2 Table. Demographic characteristics of young male participants from the general population cohort in Nairobi, by survey year

**Demographic characteristics of male participants (Nairobi)**	**2018**<br>(N=2036)	**2019**<br>(N=)
**Age group at enrolment**	**n (%)**	**n (%)**
15-17	625 (30.7)	350 (26.8)
18-19	397 (19.5)	271 (20.7)
20-22	585 (28.7)	404 (31.0)
23-24	429 (21.1)	279 (21.4)
**Highest education level completed**		
None or primary	744 (36.5)	392 (30.1)
Secondary or higher	1290 (63.4)	912 (69.9)
**Marital status**		
Never married	1782 (87.5)	1135 (87.0)
Previously (currently) married/living with partner	253 (12.4)	169 (12.9)
**Sexual experience**		
Ever had sex	1094 (53.7)	725 (55.6)
Never had sex	928 (45.6)	566 (43.4)
**Ever engaged in income generating activity, including employment**		
Yes	1096 (53.8)	662 (50.8)
No	940 (46.2)	642 (49.2)
