# Supplementary material for: Impact of DREAMS interventions on attitudes towards gender norms among adolescent girls and young women: Findings from a prospective cohort in Kenya
Source: PLOS Glob Public Health. 2024 Mar 6;4(3):e0002929. doi: 10.1371/journal.pgph.0002929 (PMC10917282; doi:10.1371/journal.pgph.0002929)
Supplement: S3 Table — (PDF) [file pgph.0002929.s006.pdf]

S3 Table. Sensitivity analysis: Estimated causal effect of DREAMS on individual attitudes towards gender norms in 2019, using different analysis approaches

Outcome, study population, and analysis approach	% Equitable attitudes in total study population	Estimated % equitable attitudes if none benefit from DREAMS (95% CI)	Estimated % equitable attitudes if all benefit from DREAMS (95% CI)	Risk Difference % (95% CI)
**SRH decision-making norms**				
**Nairobi**				
**Overall (15-22 years)**				
PS regression adjustment	89.5	87.1 (81.6, 91.9)	90.2 (87.7, 92.6)	+3.1 (-2.5, +9.0)
PS stratification	89.5	86.8 (80.7, 91.2)	90.1 (87.8, 92.7)	+3.4 (-1.7, +10.1)
PS inverse probability of treatment weighting	89.5	86.7 (81.4, 91.3)	90.0 (87.6, 92.5)	+3.5 (-2.1, +9.4)
Counterfactual	89.5	86.8 (78.3, 90.5)	89.8 (87.0, 92.1)	+3.0 (-1.5, +1.2)
**Gem**				
PS regression adjustment	89.4	88.6 (84.9, 92.0)	89.6 (86.2, 92.5)	+1.0 (-3.6, +5.6)
PS stratification	89.4	88.3 (85.0, 91.9)	89.4 (86.5, 92.3)	+1.1 (-3.6, +5.3)
PS inverse probability of treatment weighting	89.4	88.7 (85.3, 92.0)	89.6 (86.4, 92.4)	+0.8 (-3.8, +5.6)
Counterfactual	89.4	86.6 (85.2, 92.0)	89.7 (86.5, 92.5)	+1.1 (-3.3, +6.1)
**Violence-related norms**				
**Nairobi**				
PS regression adjustment	82.2	82.2 (77.0, 87.2)	82.7 (79.6, 85.7)	+0.51 (-5.3, +6.5)
PS stratification	82.2	80.4 (74.8, 86.3)	82.6 (79.8, 85.7)	+2.2 (-4.2, +8.8)
PS inverse probability of treatment weighting	82.2	81.4 (75.8, 86.8)	82.6 (79.5, 85.5)	+1.3 (-4.5, +7.2)
Counterfactual	82.2	82.2 (76.1, 87.1)	82.5 (79.5, 85.5)	+0.4 (+0.5, +0.7)
**Gem**				
PS regression adjustment	46.7	48.2 (43.1, 53.5)	44.3 (39.6, 49.0)	-3.9 (-11.7, +3.0)
PS stratification	46.7	48.5 (43.0, 54.5)	44.2 (39.4, 49.1)	-4.3 (-11.5, +2.4)
PS inverse probability of treatment weighting	46.7	48.6 (42.8, 54.2)	44.1 (38.9, 49.2)	-4.4 (-11.0, +3.2)
Counterfactual	46.7	48.4 (42.7, 53.9)	44.3 (39.6, 49.1)	-3.9 (-11.0, +3.0)
