# Supplementary material for: Impact of DREAMS interventions on attitudes towards gender norms among adolescent girls and young women: Findings from a prospective cohort in Kenya
Source: PLOS Glob Public Health. 2024 Mar 6;4(3):e0002929. doi: 10.1371/journal.pgph.0002929 (PMC10917282; doi:10.1371/journal.pgph.0002929)
Supplement: S4 Table — (PDF) [file pgph.0002929.s007.pdf]

S4 Table. Sensitivity analysis: Estimated causal effect of DREAMS intervention access ("per protocol") on individual attitudes towards gender norms in 2019

	% Equitable attitudes in total study population	Estimated % equitable attitudes if <u>none</u> benefit from DREAMS (95% CI)	Estimated % equitable attitudes if accessed <3 primary interventions (95% CI)	Risk Difference % (95% CI) *PS-adjusted*	Estimated % equitable attitudes if accessed 3+ primary interventions (95% CI)	Risk Difference % (95% CI) *PS-adjusted*
**SRH decision-making norms**						
**Nairobi**						
Overall	89.5	87.1 (81.6, 91.9)	85.3 (75.5, 92.3)	-1.8 (-13.1, +7.7)	90.7 (88.2, 93.1)	+3.6 (-1.9, +9.5)
15-17 years	88.7	87.1 (78.7, 94.2)	82.9 (71.4, 92.7)	-4.2 (-17.0, +9.4)	90.1 (86.8, 93.3)	+3.0 (-4.4, +12.1)
18-22 years	90.4	87.2 (80.9, 92.7)	90.6 (75.3, 96.2)	+3.5 (-12.9, 12.5)	91.4 (87.3, 94.9)	+4.2 (-2.4, +11.2)
**Gem**						
Overall	89.4	88.6 (84.9, 92.0)	86.4 (80.3, 91.3)	-2.5 (-9.7, +4.0)	91.7 (88.3, 94.9)	+2.8 (-2.1, +8.1)
15-17 years	85.2	84.3 (78.8, 89.8)	78.7 (68.8, 87.6)	-5.8 (-17.2, +4.7)	89.9 (84.2, 95.2)	+5.4 (-2.1, 13.5)
18-22 years	93.2	92.9 (88.7, 96.4)	93.5 (86.6, 98.4)	+0.6 (7.3, +7.6)	93.3 (89.2, 97.0)	+0.4 (-4.7, 6.2)
**Violence-related norms**						
**Nairobi**						
Overall	82.2	82.2 (77.0, 87.2)	81.9 (73.1, 88.6)	-0.3 (-10.4, +7.9)	82.9 (79.5, 86.1)	+0.7 (-5.5, +6.7)
15-17 years	82.1	85.8 (77.9, 92.1)	84.8 (73.4, 93.7)	-1.0 (-13.8, +11.1)	80.4 (75.9, 84.6)	-5.4 (-13.3, +3.3)
18-22 years	82.2	78.0 (70.8, 84.7)	78.5 (60.8, 89.1)	+0.5 (-17.0, +13.1)	85.8 (80.7, 90.2)	+7.8 (-0.6, +16.0)
**Gem**						
Overall	46.7	48.2 (43.1, 53.5)	40.5 (32.0, 49.8)	-7.7 (-17.9, +3.1)	46.1 (40.2, 52.3)	-2.1 (-10.1, +5.9)
15-17 years	41.6	45.5 (38.1, 53.5)	34.2 (23.9, 45.5)	-10.5 (-23.9, +2.9)	40.7 (32.0, 49.2)	-4.1 (-15.8, +7.3)
18-22 years	51.3	52.2 (44.9, 60.2)	46.2 (34.3, 59.6)	-5.1 (-18.8, +10.3)	51.1 (43.8, 59.0)	-0.3 (-11.3, +10.2)
