# Supplementary material for: Impact of DREAMS interventions on attitudes towards gender norms among adolescent girls and young women: Findings from a prospective cohort in Kenya
Source: PLOS Glob Public Health. 2024 Mar 6;4(3):e0002929. doi: 10.1371/journal.pgph.0002929 (PMC10917282; doi:10.1371/journal.pgph.0002929)
Supplement: S1 Text — A) Standardised factor loadings and factor correlations for an exploratory four-factor model of GEM Scale responses in 2017, among AGYW in Nairobi (n = 892) B) Standardised factor loadings following confirmatory factor analysis in Gem (2018), comparing two and four factor models with and without reducing scale items. C) Scree plot of eigenvalues after exploratory factor analysis (Nairobi 2017). (PDF) [file pgph.0002929.s008.pdf]

S1 Text. Validation of GEM Scale

We validated the modified GEM scale using factor analysis, which aims to identify 'latent factors' using the common variance among observed variables.(1) First, we applied investigator-driven exploratory factor analysis (EFA) to the 2017 Nairobi cohort data, to identify the dimensions of gender norms measured by the scale. We then used confirmatory factor analysis (CFA) with the 2018 Gem cohort data to estimate model fit.

At the exploratory stage, individuals who refused or responded "don't know" to any items were excluded. Equitably-phrased items were reverse-coded so that a higher score always indicated support for equitable norms (agreement with equitable statements or disagreement with inequitable statements).

Factors were extracted using the polychoric correlation matrix.(1) The number of factors to retain was determined by examination of the scree plot and consideration of the number of factors with an eigenvalue above one.(2) Items that were poor measures of the factor (uniqueness >0.7) were dropped. Following oblique rotation, factors were then interpreted as dimensions of gender norms.(3)

Next we fit three CFA models to the 2018 Gem data, using weighted least squares with mean and variance adjustment: Model 1 specified the factor structure and subset of items identified through EFA; Model 2 specified the two factors from the original GEM scale with the same subset of items (identified through EFA); and Model 3 specified the tw07/02/2024 22:59:00o factor structure with all items included.(4)

Model fit was compared across relative and absolute measures including the Comparative Fit Index (>0.95 indicates acceptable fit), Tucker-Lewis Index (>0.95 acceptable) and Root Mean Square Error of Approximation (<0.08 acceptable).(5) The dimensions of gender norms identified by the best-fitting model were taken as outcomes, provided they had adequate internal reliability (ordinal alpha >=0.70).

EFA resulted in four factors with eigenvalues above 1, consistent with the scree plot inflection point (S3 Figure).(2) Seven items with uniqueness >0.7 were dropped, leaving a subset of 16 items. We interpreted the factors as representing norms relating to 'SRH decision-making,' 'violence,' 'need for sex,' and 'initiation of sex.'

The four-factor structure in Model 1 provided the best fit, indicated by high factor loadings and superior fit statistics obtained from the CFA models (Table S3b). We therefore used this model to define the outcomes. However two factors (need for sex and initiation of sex) were excluded as they were measured with few items and had low reliability (ordinal alpha = 0.67, 0.62). Score distributions for the retained dimensions are shown in S4.

1. Fabrigar LR, Wegener DT, MacCallum RC, Strahan EJ. Evaluating the use of exploratory factor analysis in psychological research. Psychological Methods. 1999;4(3):272-99.
2. Cattell RB, Vogelmann S. A Comprehensive Trial Of The Scree And Kg Criteria For Determining The Number Of Factors. Multivariate Behav Res. 1977;12(3):289-325.
3. Hendrickson AE, White PO. PROMAX: A quick method for rotation to oblique simple structure. British Journal of Statistical Psychology. 1964;17(1):65-70.
4. Flora DB, Curran PJ. An Empirical Evaluation of Alternative Methods of Estimation for Confirmatory Factor Analysis With Ordinal Data. Psychological Methods. 2004;9(4):466-91.
5. Streiner DL. Building a Better Model: An Introduction to Structural Equation Modelling. The Canadian Journal of Psychiatry. 2006;51(5):317-24.

Table A. Standardised factor loadings and factor correlations for an exploratory four-factor model of GEM Scale responses in 2017, among AGYW in Nairobi (n=892)

**Retained GEM Scale Items (n=16)**	Standardised factor loadings[1]				Uniqueness
A couple should decide together if they want to have children.	**0.86**	-0.02	-0.15	-0.02	0.34
In my opinion, a woman can suggest using condoms just like a man can.	**0.60**	0.06	0.001	0.01	0.61
If a man gets a woman pregnant, the child is the responsibility of both.	**0.70**	0.001	0.01	0.01	0.51
A man and a woman should decide together what type of contraceptive to use.	**0.77**	-0.05	-0.04	0.07	0.46
A man and woman should decide together whether to use a condom.	**0.71**	-0.03	0.05	0.03	0.48
There are times when a woman deserves to be beaten.	0.05	**0.63**	0.06	-0.003	0.54
A woman should tolerate violence in order to keep her family together.	-0.15	**0.66**	-0.05	-0.01	0.64
If a woman cheats on a man, it is okay for him to hit her.	-0.07	**0.74**	-0.12	0.14	0.51
If someone insults a man he should defend his reputation with force if he has to.	-0.02	**0.60**	0.03	0.07	0.62
A man should be outraged if his wife/partner asks him to use a condom.	0.28	**0.46**	0.1	-0.04	0.53
It is okay for a man to hit his wife if she won't have sex with him.	0.31	**0.42**	0.16	-0.15	0.51
It is the man who decides what type of sex to have.	-0.04	0.03	**0.76**	-0.03	0.44
Men need sex more than women do.	-0.15	-0.05	**0.77**	0.01	0.49
You don't talk about sex, you just do it.	0.13	0.03	**0.56**	-0.04	0.61
A woman who has sex before she marries does not deserve respect.	0.02	0.09	-0.07	**0.91**	0.17
A woman should not initiate sex.	0.04	-0.07	0.24	**0.50**	0.65
Factor Interpretations:	*SRH decision-making*	*Violence*	*Need/ Readiness for sex*	*Initiation of sex*	

[1]Bolding indicates loadings >0.40; 'Don't know' treated as missing

Figure. Scree plot of eigenvalues after exploratory factor analysis (Nairobi 2017)

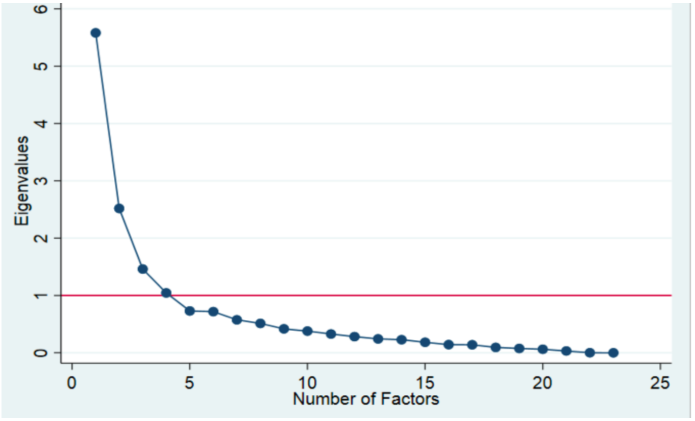

Table B. Standardised factor loadings following confirmatory factor analysis in Gem (2018), comparing 2 and 4 factor models with and without reducing scale items

	**Model 1: Four factor model (16 items)**				**Model 2: Two factors model (16 items)**		**Model 3: Two factor model (All 23 items)**	
**a. Gem Scale Items**	SRH decision-making	Violence	Need for sex	Initiation of sex	Equitable	Inequitable	Equitable	Inequitable
A couple should decide together if they want to have children.	0.79 (0.02)				0.78 (0.02)		0.78 (0.02)	
In my opinion, a woman can suggest using condoms just like a man can.	0.56 (0.02)				0.56 (0.02)		0.57 (0.02)	
If a man gets a woman pregnant, the child is the responsibility of both.	0.84 (0.01)				0.84 (0.01)		0.84 (0.01)	
A man and a woman should decide together what type of contraceptive to use.	0.85 (0.02)				0.85 (0.02)		0.84 (0.02)	
A man and woman should decide together whether to use a condom.	0.67 (0.02)				0.67 (0.02)		0.68 (0.02)	
A man should know what his partner likes during sex.							0.36 (0.06)	
There are times when a woman deserves to be beaten.		0.65 (0.02)				0.64 (0.02)		0.60 (0.02)
A woman should tolerate violence in order to keep her family together.		0.35 (0.03)				0.36 (0.03)		0.36 (0.03)
If a woman cheats on a man, it is okay for him to hit her.		0.59 (0.02)				0.36 (0.03)		0.55 (0.02)
If someone insults a man he should defend his reputation with force if he has to.		0.59 (0.03)				0.58 (0.03)		0.54 (0.03)
A man should be outraged if his wife/partner asks him to use a condom		0.58 (0.03)				0.58 (0.03)		0.57 (0.03)
It is okay for a man to hit his wife if she won't have sex with him.		0.73 (0.02)				0.73 (0.02)		0.74 (0.02)
It is the man who decides what type of sex to have			0.72 (0.08)			0.29 (0.06)		0.31 (0.05)
Men need sex more than women do			0.64 (0.07)			0.15 (0.05)		0.18 (0.05)
You don't talk about sex, you just do it			0.53 (0.08)			0.23 (0.06)		0.27 (0.06)
A woman who has sex before she marries does not deserve respect.				0.52 (0.08)		0.15 (0.05)		0.21 (0.05)
A woman should not initiate sex				0.87 (0.12)		0.23 (0.05)		0.29 (0.05)
Men are always ready to have sex								0.19 (0.05)
Women who carry condoms are "easy."								0.38 (0.03)
It is a woman's responsibility to avoid getting pregnant.								-0.09 (0.03)
Only when a woman has a child is she a real woman.								0.57 (0.02)
A real man produces a male child.								0.76 (0.02)
A man using violence against his wife is a private matter that shouldn't be discussed outside the couple.								0.33 (0.03)

**b. Fit indices (Robust)**	**Model 1**				**Model 2**		**Model 3**	
Chi 2 (degrees of freedom)	481.7 (98)				692.6 (103)		1980.3 (229)	
Comparative fit index (CFI)	0.95				0.92		0.83	
Tucker-Lewis Index (TLI)	0.94				0.91		0.81	
Root Mean Square Error of Approximation (RMSEA)	0.07				0.08		0.09	
Standardised Root Mean Square Residual (SRMR)	0.06				0.10		0.12	
**c. Factor correlations**								
Factor 1	1				1		1	
Factor 2	0.39	1			0.4	1	0.4	1
Factor 3	-0.02	0.3	1					
Factor 4	0.05	0.2	0.5	1				
